# Circadian Gene Networks and Transcriptome Oscillations in Prostate Cancer: Insights From RNA Sequencing and Implications for Chronotherapy

**DOI:** 10.7759/cureus.113586

**Published:** 2026-07-29

**Authors:** Ivan Bivolarski, Martin Dimitrov, Hristo Dechev

**Affiliations:** 1 Chronotherapy and Cancer Research, Integrated Oncology Centre-Burgas, Burgas, BGR; 2 Surgery, Klinik für Allgemeine, Gefäß- und Viszerale Chirurgie, Kreiskrankenhaus Greiz, Greiz, DEU; 3 Medical Oncology, Integrated Oncology Centre-Burgas, Burgas, BGR

**Keywords:** alternative splicing, androgen receptor (ar), chronotherapy, circadian rhythms, clock genes, prostate cancer, rna-seq, spatial transcriptomics

## Abstract

Prostate cancer is increasingly recognized as a disease influenced not only by genetic and molecular alterations but also by disruption of circadian regulatory networks. Advances in RNA sequencing (RNA-Seq) have enabled transcriptome-wide investigation of temporal gene expression patterns, revealing complex interactions between core clock genes, androgen receptor signaling, alternative splicing, and metabolic pathways. Emerging evidence suggests that circadian dysregulation contributes to prostate cancer progression through alterations in gene expression, transcript isoform remodeling, and treatment resistance mechanisms. In particular, RNA-Seq studies have provided new insights into the relationship between clock gene networks and androgen receptor signaling, as well as the potential role of alternative splicing in the development of aggressive disease phenotypes. Recent bioinformatic approaches have further enabled the analysis of temporal patterns within large transcriptomic datasets lacking time-of-collection information. These advances have generated growing interest in chronotherapy, in which treatment timing may be optimized according to biological rhythms. Although clinical implementation remains limited, circadian transcriptomics offers a promising framework for understanding prostate cancer biology and developing more individualized therapeutic strategies. This review summarizes current evidence regarding circadian regulation in prostate cancer, with particular emphasis on RNA-Seq-derived insights into clock gene networks, transcriptome oscillations, alternative splicing, and potential chronotherapeutic applications.

## Introduction and background

Clinical significance of prostate cancer

Prostate cancer is one of the most frequently diagnosed malignancies and a leading cause of cancer-related mortality among men worldwide. According to the latest Global Cancer Observatory estimates, approximately 1.55 million new cases and 420,000 deaths occurred globally in 2024 [1]. Despite major advances in screening, molecular classification, and systemic treatment, substantial clinical heterogeneity persists, particularly in advanced disease, where treatment resistance remains a major challenge. These observations underscore the need to identify additional biological determinants of tumor behavior and therapeutic response.

Circadian regulation and cancer

Cancer has traditionally been studied in terms of genetic, molecular, and spatial heterogeneity. Increasing attention is now being directed toward temporal heterogeneity and the role of circadian regulation in tumor biology. The circadian system comprises a central pacemaker and peripheral molecular clocks that coordinate physiological processes through approximately 24-hour oscillations in gene expression. At the molecular level, these rhythms are generated by interconnected transcription-translation feedback loops in which CLOCK and BMAL1 activate the expression of PERIOD (PER1-3) and CRYPTOCHROME (CRY1-2) genes, whose protein products subsequently inhibit CLOCK-BMAL1 activity. Additional regulatory loops involving REV-ERB and ROR proteins contribute to the stability and precision of these oscillations [2].

Circadian rhythms influence DNA repair, cell-cycle regulation, metabolism, hormonal signaling, and immune activity. Their disruption may therefore promote malignant transformation and tumor progression by impairing cellular homeostasis [2]. Consistent with this biological association, the International Agency for Research on Cancer has classified night shift work involving circadian disruption as probably carcinogenic to humans [3].

The prostate circadian clock

The prostate contains an intrinsic molecular clock whose activity is closely connected to androgen-dependent physiology. Altered expression of PER1, PER2, BMAL1, CLOCK, and CRY1 has been reported in experimental and clinical studies of prostate cancer, although the direction and functional significance of these changes have not been consistent across all models and disease stages [4,5]. Circadian pathways also interact with androgen receptor (AR) signaling, a central driver of prostate cancer development and progression. This bidirectional relationship may influence cellular proliferation, apoptosis, DNA-damage responses, and treatment resistance [4,5].

RNA sequencing (RNA-Seq) and circadian transcriptomics

Traditional approaches to studying circadian regulation in cancer, including quantitative reverse-transcription polymerase chain reaction and immunohistochemistry, generally examine a limited number of candidate genes or proteins. In contrast, RNA-Seq enables transcriptome-wide analysis, providing information on gene- and transcript-level expression, previously unrecognized transcripts, alternative splicing, and AR splice variants. It therefore offers a broader framework for identifying circadian-associated molecular alterations that may not be captured by targeted methods [6].

RNA-Seq and related computational analyses suggest that circadian disruption may affect downstream pathways involved in metabolism, immune regulation, DNA repair, and alternative mRNA splicing [4,5,7]. Computational methods have further expanded the analysis of temporal transcriptomic patterns. CYCLOPS can infer the relative circadian order of human samples collected without recorded sampling times, whereas MetaCycle integrates multiple algorithms for rhythm detection and JTK_CYCLE identifies rhythmic patterns in time-series datasets using a nonparametric approach [8-10]. However, their performance depends on factors such as sampling density, cohort size, signal strength, and the preservation of rhythmicity within the examined tissue [8-10].

Chronotherapy and current knowledge gaps

Chronotherapy seeks to align treatment administration with biological rhythms to improve efficacy or reduce toxicity. Circadian regulation may influence drug pharmacokinetics-including absorption, distribution, metabolism, and elimination-as well as pharmacodynamic responses in tumor and normal tissues. Consequently, the same treatment may produce different therapeutic and adverse effects depending on the time of administration [11]. This concept is potentially relevant to prostate cancer therapies, although prospective evidence supporting specific circadian treatment schedules remains limited [5].

Current evidence is constrained by small and heterogeneous cohorts, conflicting findings regarding clock-gene expression, and frequent reliance on single-time-point sampling [4,5]. Longitudinal and time-resolved prostate cancer transcriptomic datasets remain scarce, and validated biomarkers of individual circadian phase are not yet available for routine clinical use. Interindividual variability and the absence of prospective treatment-timing trials further limit translation into clinical practice [4,5].

Aim and scope of the review

This review critically synthesizes current evidence on circadian regulation in prostate cancer, with particular emphasis on clock-gene networks, AR signaling, RNA-Seq-derived transcriptomic alterations, alternative splicing, and emerging chronotherapeutic approaches. Its novelty lies in integrating circadian biology, prostate cancer transcriptomics, splice-variant regulation, computational rhythm analysis, and treatment timing within a unified framework. By identifying methodological limitations and unresolved translational questions, the review also outlines priorities for incorporating temporal biology into precision oncology.

Literature search strategy

A narrative literature search was conducted using PubMed, Scopus, Web of Science, and Google Scholar, covering publications available from database inception through July 2026. The principal search terms included “prostate cancer,” “circadian rhythm,” “circadian clock,” “clock genes,” “BMAL1,” “CLOCK,” “PER1,” “PER2,” “CRY1,” “androgen receptor,” “RNA sequencing,” “RNA-Seq,” “circadian transcriptomics,” “alternative splicing,” “androgen receptor variants,” “CYCLOPS,” “MetaCycle,” “JTK_CYCLE,” “chronotherapy,” and “treatment timing.” These terms were combined using the Boolean operators “AND” and “OR” to identify studies addressing the relationships among circadian regulation, transcriptomic alterations, and therapeutic implications in prostate cancer.

Peer-reviewed original studies, clinically relevant translational investigations, methodological publications, and narrative or systematic reviews published in English were considered eligible. Priority was given to recent publications, seminal mechanistic studies, and articles directly addressing circadian biology, androgen receptor signaling, RNA-Seq, alternative splicing, computational rhythm analysis, or chronotherapy in prostate cancer. Duplicate publications, conference abstracts without a corresponding full-text article, non-English-language reports, and studies without clear relevance to the review objectives were excluded. Reference lists of selected articles were also examined to identify additional pertinent publications. The evidence was synthesized narratively and organized thematically; because this was a narrative review, no formal risk-of-bias assessment, meta-analysis, or the Preferred Reporting Items for Systematic Reviews and Meta-Analyses (PRISMA) flow diagram was undertaken.

## Review

The core clock machinery and the prostate cancer transcriptome

The development and progression of prostate cancer are associated not only with genetic and epigenetic alterations but also with disruption of circadian regulatory networks. Experimental studies have identified altered expression and functional activity of individual clock components, while epidemiological and translational investigations suggest that circadian gene variation may contribute to interindividual differences in disease susceptibility and outcome [12-17]. Increasing evidence also supports interactions among circadian pathways, AR signaling, DNA-damage responses, metabolism, and therapeutic resistance.

Alterations in clock gene networks

The molecular circadian clock is based on interconnected transcription-translation feedback loops involving CLOCK, BMAL1 (ARNTL), PER1-3, and CRY1-2. In healthy tissues, these components coordinate rhythmic processes related to cellular proliferation, metabolism, DNA repair, apoptosis, and tissue homeostasis. In prostate cancer, however, individual clock genes may acquire distinct and context-dependent functions.

PER1 appears to exert tumor-suppressive effects in prostate cancer. Experimental suppression of PER1 has been associated with reduced apoptosis and altered expression of genes involved in cell-cycle regulation and DNA-damage responses, whereas its restoration may inhibit malignant cell growth [12]. Dysregulation of PER2, BMAL1, and CLOCK has also been observed in human prostate cancer cells. In one experimental model, CLOCK and PER2 expression was reduced, whereas BMAL1 expression was increased relative to nonmalignant prostate cells. Melatonin treatment partially restored coordinated clock-gene expression and suppressed cellular proliferation, suggesting that circadian circuitry remains therapeutically modifiable in at least some prostate cancer models [13].

CRY1 may have a contrasting, context-dependent role. In prostate cancer, CRY1 has been identified as an AR-regulated, protumorigenic factor that supports DNA-damage repair and may promote survival under genotoxic stress. Its expression and function appear to become particularly relevant in advanced disease, illustrating that not all clock components behave uniformly as tumor suppressors [14].

Population-based studies provide additional, although not entirely consistent, evidence. Associations between selected circadian gene variants and prostate cancer susceptibility have been reported [15]. However, an analysis of genetic variation across core circadian genes did not demonstrate a strong and consistent association with fatal prostate cancer, despite several nominal gene-level associations [16]. These differences highlight the heterogeneity of the available evidence and suggest that the effects of circadian genes may depend on genetic background, tumor context, disease stage, and environmental exposure.

**Figure 1:**
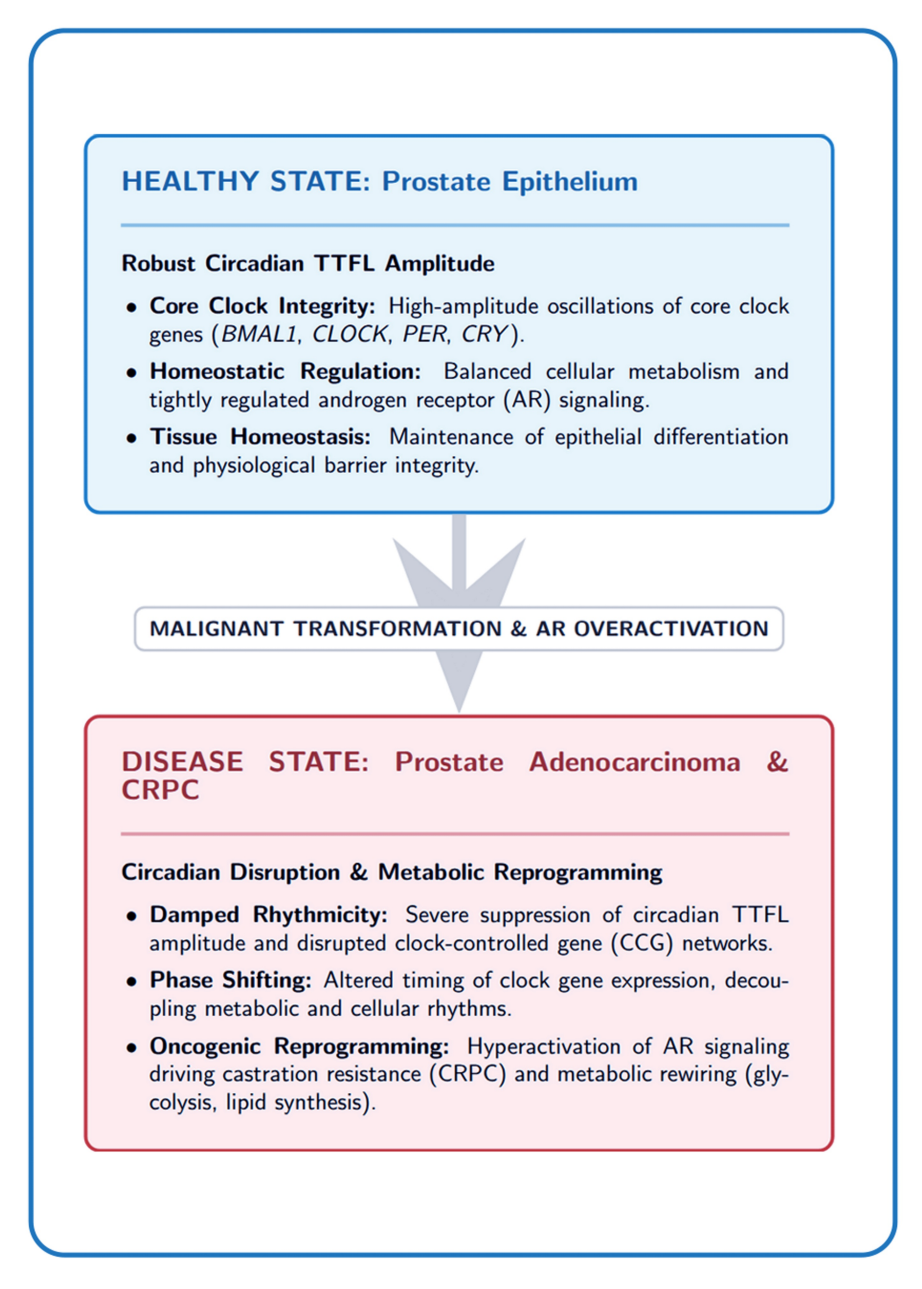
Circadian disruption during prostate cancer progression AR: androgen receptor Circadian disruption during prostate cancer progression. Progression from healthy prostate tissue to advanced prostate cancer is associated with loss of circadian rhythmicity, metabolic reprogramming, and enhanced AR signaling, contributing to castration resistance. This illustration was created by the authors using Adobe Photoshop (Adobe Inc.; San Jose, California, USA)

A conceptual overview of the transition from a circadian-regulated prostate epithelium to prostate adenocarcinoma and castration-resistant prostate cancer (CRPC) is presented in Figure 1. Rather than reflecting uniform suppression or activation of the entire clock network, disease progression may involve selective remodeling of individual clock components, altered phase relationships, and loss of coordinated rhythmic output [12-17].

Recent evidence further suggests that circadian disruption may affect prostate cancer through multiple interconnected mechanisms. These include altered AR signaling, enhanced DNA damage repair, metabolic adaptation, and impaired coordination between proliferation and apoptosis. Such effects may become progressively more important during the transition to treatment-resistant disease [14,17].

Interaction between circadian pathways and AR signaling

The AR is a central regulator of prostate cancer development and progression. Experimental and translational studies suggest a bidirectional relationship between AR signaling and the circadian clock: clock components may influence AR transcriptional activity, whereas androgen signaling can modify the expression or function of circadian regulators [14,17]. This interaction may be especially relevant in CRPC, in which persistent AR activity remains a major driver despite castrate levels of circulating androgens.

Circadian regulation may also intersect with DNA-damage response pathways that are influenced by hormonal signaling. However, evidence supporting this connection should be interpreted carefully because much of it derives from broader studies of hormone-dependent cancers rather than time-resolved prostate cancer models [18]. Metabolic regulation represents another potential interface. Circadian clocks coordinate lipid and energy metabolism, while prostate cancer cells undergo extensive metabolic reprogramming during progression. These observations provide a biological rationale for investigating BMAL1- and CLOCK-associated metabolic pathways, although direct prostate cancer-specific evidence remains limited [17,19].

Overall, current findings support a relationship between clock disruption and AR-driven prostate cancer biology. Nevertheless, the direction and magnitude of this relationship appear to vary across experimental models and disease stages, and prospective clinical validation is still lacking.

Deciphering transcriptome oscillations via RNA-Seq

RNA-Seq has expanded the study of circadian biology by enabling transcriptome-wide assessment of gene expression, transcript isoforms, and pathway-level alterations. Circadian transcriptomic atlases demonstrate that rhythmic gene expression is highly tissue-specific and extends far beyond the canonical clock genes [20]. Importantly, these atlases provide a general framework for circadian transcriptomics but do not constitute direct evidence of rhythmic transcription in prostate cancer.

**Figure 2:**
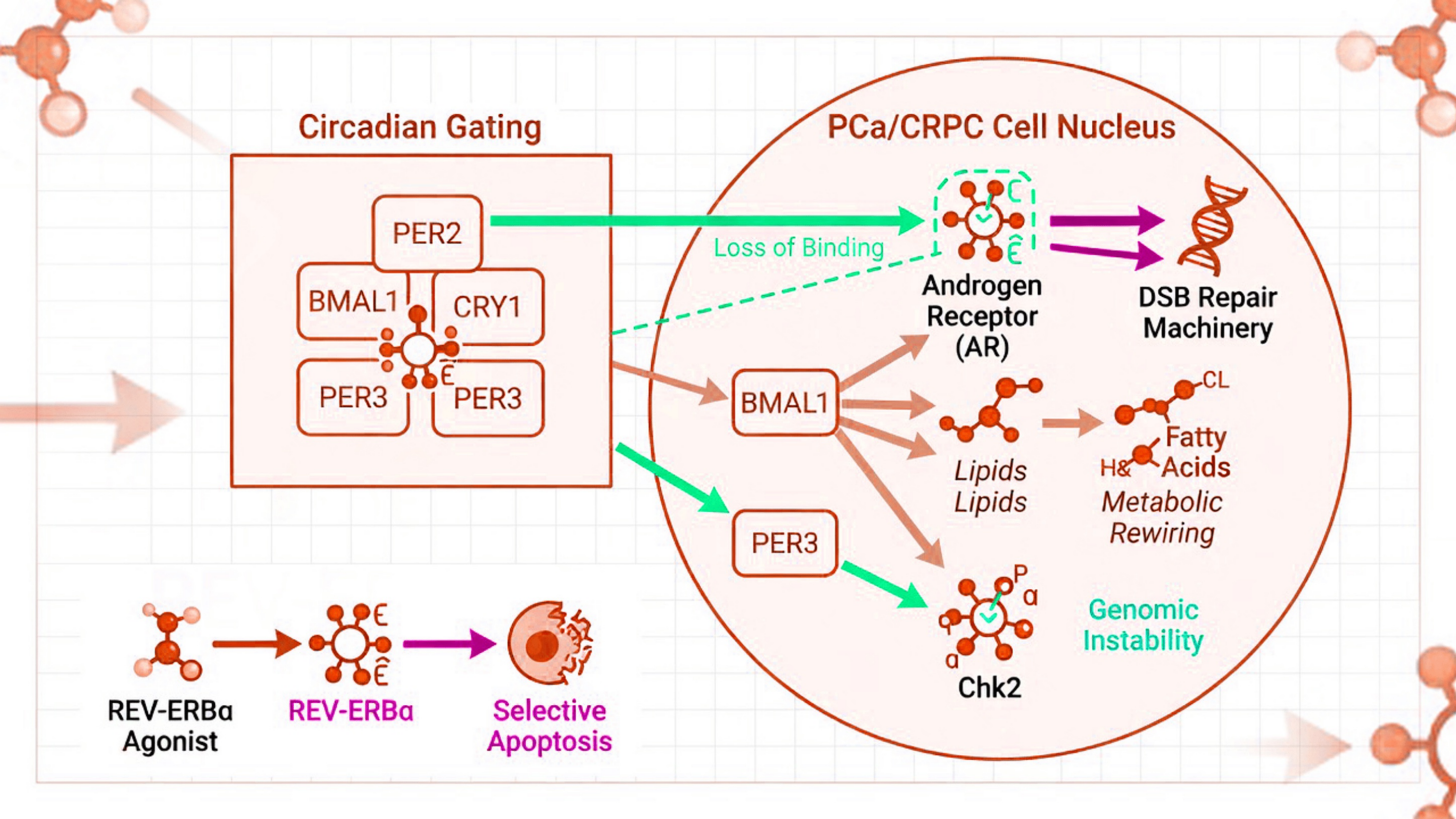
Mechanistic model of circadian dysregulation in prostate cancer AR: androgen receptor Disruption of core circadian clock components alters AR signaling, DNA damage repair pathways, cellular metabolism, and genomic stability. Pharmacological activation of REV-ERBα may counteract these oncogenic processes and promote tumor cell apoptosis. This illustration was created by the authors using Adobe Photoshop (Adobe Inc.; San Jose, California, USA)

In prostate tumors, most available RNA-Seq datasets were generated from samples obtained at a single recorded or unrecorded time point. Consequently, they can identify altered clock-gene expression and circadian-associated pathways but cannot independently establish genuine 24-hour oscillation. Selective changes in amplitude, phase, or rhythmic coordination therefore remain biologically plausible models that require confirmation using serially collected or computationally ordered samples. Figure 2 illustrates this proposed transition from coordinated physiological oscillation to selective temporal remodeling during prostate cancer progression.

This distinction is methodologically important. Differences between tumor and normal tissue may reflect disease biology, sampling time, or an interaction between the two. Accordingly, claims of altered circadian oscillation should be distinguished from observations based solely on differential expression in cross-sectional datasets.

Alternative Splicing and Transcript Isoform Remodeling

Beyond measuring total gene expression, RNA-Seq provides detailed information on transcript structure and alternative splicing. Alternative splicing enables a single gene to generate multiple transcript isoforms with distinct or opposing biological functions and represents an important source of molecular heterogeneity in cancer.

Components of the splicing machinery are themselves subject to circadian regulation. Experimental evidence indicates that both the molecular clock and external timing cues can influence alternative exon usage and transcript isoform production [21]. Disruption of circadian coordination could therefore affect not only transcript abundance but also transcript structure, potentially modifying proteins involved in proliferation, metabolism, apoptosis, and therapeutic response.

Alternative splicing is particularly relevant in advanced prostate cancer because it contributes to the generation of constitutively active AR splice variants. Transcriptomic analyses demonstrate that these variants may regulate partially distinct transcriptional programs and promote adaptation under androgen-deprived conditions [22]. Experimental cotargeting of AR splice variants and mTOR signaling has further demonstrated the functional relationship between splice-variant activity, survival pathways, and treatment resistance in CRPC models [23].

Among the AR variants, AR-V7 has the strongest clinical evidence. In a prospective study of men with metastatic CRPC, detectable AR-V7 in circulating tumor cells was associated with no prostate-specific antigen response to either enzalutamide or abiraterone, compared with response rates of 53% and 68%, respectively, among AR-V7-negative patients. AR-V7 positivity was also associated with shorter progression-free and overall survival [24]. These findings establish the clinical relevance of transcript isoform remodeling, although a direct circadian mechanism underlying AR-V7 generation has not yet been demonstrated.

Overall, RNA-Seq indicates that prostate cancer progression involves changes at both gene-expression and transcript-isoform levels. Interactions between circadian regulation and alternative splicing remain an emerging hypothesis that requires validation through time-resolved experimental studies.

Methodological landscape: challenges in circadian transcriptomics

The study of circadian biology in cancer presents important methodological challenges. Most clinical transcriptomic datasets were generated without recording the time of tissue collection. Despite the availability of large-scale resources such as the Cancer Genome Atlas and the International Cancer Genome Consortium, the temporal dimension of gene regulation, therefore, remains largely invisible.

In routine clinical practice, tissue samples are collected according to institutional schedules rather than circadian considerations. Transcriptomic differences between samples may consequently reflect variation in biological timing in addition to underlying tumor biology. Because many genes exhibit circadian oscillations, missing temporal metadata may confound differential gene-expression analyses and obscure biologically relevant temporal patterns.

Additional limitations include sparse sampling, small cohort sizes, differences across sequencing platforms, tumor heterogeneity, batch effects, and variability in individual circadian phases. Clock time is also not equivalent to internal biological time because chronotype, sleep patterns, light exposure, medication, and systemic illness may shift circadian phase.

Chronobioinformatic Approaches

Computational methods offer a potential means of recovering temporal information from existing datasets. JTK_CYCLE is a nonparametric algorithm designed to detect rhythmic components within time-series data. It is computationally efficient and relatively robust but generally requires adequately sampled temporal observations and predefined candidate periods [10].

Other approaches focus on estimating internal circadian phase from transcriptomic signatures. Machine-learning analysis has identified compact gene sets capable of monitoring circadian timing in human blood, although performance may be affected by tissue specificity and clinical context [25]. Blood transcriptome-based biomarkers have similarly demonstrated that circadian phase can be estimated from a limited number of samples, supporting the feasibility of molecular phase assessment in humans [26]. Additional computational frameworks can infer circadian state directly from gene-expression profiles and may facilitate the temporal reanalysis of archived datasets [27].

**Figure 3:**
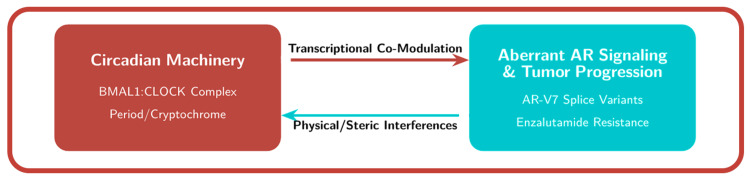
Bidirectional interaction between circadian clock components and androgen receptor signaling AR: androgen receptor Circadian clock regulators modulate AR activity, while aberrant AR signaling reciprocally disrupts circadian clock function. This bidirectional feedback loop may contribute to AR-V7 expression, enzalutamide resistance, and prostate cancer progression. This illustration was created by the authors using Adobe Photoshop (Adobe Inc.; San Jose, California, USA)

These methods have important limitations. Their accuracy depends on signal strength, training population, tissue type, sampling conditions, and preservation of rhythmicity in diseased tissue. A model developed in blood or healthy organs may not transfer directly to heterogeneous prostate tumors. Computationally inferred phase should therefore be regarded as an estimate rather than a substitute for prospectively collected time-resolved data. Figure 3 summarizes the proposed bidirectional interaction between circadian clock components and AR signaling and its potential contribution to AR-V7 expression, enzalutamide resistance, and prostate cancer progression.

Chronotherapeutic Implications

Chronotherapy seeks to align treatment administration with biological rhythms to improve efficacy, reduce toxicity, or both. Circadian regulation may influence drug absorption, distribution, metabolism, elimination, cellular uptake, target availability, DNA repair, and tissue sensitivity. Treatment administered at different biological times may therefore produce different therapeutic and adverse effects [11].

Chronomodulated Chemotherapy

Docetaxel remains an important treatment option for advanced prostate cancer. Its activity depends on disruption of microtubule dynamics and mitotic progression, both of which may vary with cellular timing. However, direct prospective evidence establishing an optimal circadian administration time for docetaxel in prostate cancer is currently insufficient.

The broader field of cancer chronotherapeutics provides experimental and clinical evidence that treatment timing can influence efficacy and toxicity, although the direction of benefit varies by drug, tumor type, sex, and individual circadian phenotype [28]. Enzymes involved in drug metabolism and transport are also subject to circadian regulation, supporting the biological rationale for time-adjusted administration schedules [11,28].

Systems chronotherapeutics integrates circadian biology with pharmacokinetic, pharmacodynamic, and mathematical modeling to develop individualized treatment schedules. This approach may be more informative than assigning all patients to a fixed morning or evening schedule because internal circadian phase can differ substantially between individuals [29].

Chronotherapy and AR Signaling

AR pathway inhibitors, including enzalutamide, abiraterone, apalutamide, and darolutamide, are central components of advanced prostate cancer treatment. Because AR signaling interacts with circadian regulatory pathways, temporal variation in AR-related transcriptional activity could theoretically influence therapeutic response [14,17].

Circadian mechanisms also regulate metabolism, DNA repair, cell-cycle activity, and drug-processing pathways, providing several potential routes through which treatment timing might affect AR-directed therapy. Nevertheless, there is currently insufficient prospective clinical evidence to recommend specific administration times for individual AR pathway inhibitors. The possibility of circadian optimization should therefore be presented as a testable translational hypothesis rather than an established clinical strategy.

More broadly, preclinical and translational studies indicate that targeting circadian regulators or aligning treatment with circadian biology may create new opportunities in cancer therapy [30]. Future prostate cancer trials should incorporate precise administration times, measures of individual circadian phase, longitudinal molecular sampling, and clinically meaningful efficacy and toxicity endpoints.

Limitations

This review has several limitations. First, much of the evidence concerning circadian regulation in prostate cancer derives from preclinical experiments, cross-sectional transcriptomic analyses, and computational modeling rather than prospective clinical trials. Second, many large genomic datasets lack precise information regarding tissue-collection time, sleep-wake behavior, chronotype, light exposure, and other factors required to interpret circadian phase. Third, findings across clock genes are heterogeneous and sometimes conflicting, reflecting differences in experimental models, patient populations, disease stages, and analytical methods.

In addition, evidence linking circadian regulation to alternative splicing and AR-variant formation remains largely indirect. Although AR-V7 is a clinically relevant biomarker of resistance, a causal circadian mechanism for its generation has not been established. Similarly, clinical evidence supporting chronotherapy in prostate cancer remains insufficient, and standardized treatment-timing protocols are not currently available.

Finally, this article was designed as a narrative rather than a systematic review. Study selection and evidence synthesis may therefore be subject to selection bias, and no formal risk-of-bias assessment, meta-analysis, or quantitative grading of evidence certainty was performed. These limitations emphasize the need for prospective, time-resolved, and independently validated investigations.

Future perspectives

Future research should focus on the prospective characterization of circadian regulation in prostate cancer using standardized and time-resolved study designs. Longitudinal transcriptomic profiling, repeated liquid biopsy sampling, and the integration of genomic, epigenomic, proteomic, and metabolomic data may help distinguish stable tumor-associated alterations from time-dependent molecular variation. Such approaches could also support the development of validated circadian biomarkers reflecting individual biological phase, tumor rhythmicity, and treatment response.

Wearable devices capable of continuously monitoring rest-activity cycles, sleep, body temperature, and other physiological parameters may provide complementary information on systemic circadian organization. Integration of these data with blood-based biomarkers and tumor transcriptomic profiles could facilitate individualized assessment of circadian phase. Computational modeling and machine-learning methods may subsequently assist in identifying clinically relevant temporal patterns and optimizing treatment schedules [25-27,29].

The clinical implementation of precision chronomedicine will require prospective trials incorporating predefined administration times, standardized circadian measurements, and clinically relevant efficacy and toxicity endpoints. Circadian biomarkers and AI-guided scheduling should be evaluated within established precision-oncology frameworks rather than as independent approaches. Until such strategies undergo analytical and clinical validation, their use in prostate cancer should remain investigational.

## Conclusions

Emerging evidence indicates that circadian regulation plays an important role in prostate cancer biology. RNA-Seq studies have demonstrated that alterations in clock gene networks are associated with changes in gene expression, alternative splicing, metabolism, and AR signaling. Advances in transcriptomics and computational approaches have enabled the investigation of temporal patterns within large clinical datasets, providing new insights into the relationship between circadian disruption and tumor progression. These findings have also generated interest in chronotherapy, in which treatment timing may be optimized according to biological rhythms.

Despite promising preclinical and translational data, the clinical implementation of chronotherapeutic strategies in prostate cancer remains limited. Future research should focus on prospective clinical studies, validation of noninvasive circadian biomarkers, and integration of temporal information into precision oncology frameworks. In conclusion, circadian transcriptomics represents a growing area of research that may improve understanding of prostate cancer biology and support the development of more individualized therapeutic strategies. Continued advances in transcriptomic profiling and chronobiology may further facilitate the integration of temporal principles into precision oncology.
